# Comparative Analysis of Homologous Sequences of *Saccharum officinarum* and *Saccharum spontaneum* Reveals Independent Polyploidization Events

**DOI:** 10.3389/fpls.2018.01414

**Published:** 2018-09-25

**Authors:** Anupma Sharma, Jinjin Song, Qingfan Lin, Ratnesh Singh, Ninfa Ramos, Kai Wang, Jisen Zhang, Ray Ming, Qingyi Yu

**Affiliations:** ^1^Texas A&M AgriLife Research and Extension Center at Dallas, Texas A&M University System, Dallas, TX, United States; ^2^FAFU and UIUC-SIB Joint Center for Genomics and Biotechnology, Fujian Provincial Key Laboratory of Haixia Applied Plant Systems Biology, Key Laboratory of Genetics, Breeding and Multiple Utilization of Crops, Ministry of Education, Haixia Institute of Science and Technology, College of Life Science, Fujian Agriculture and Forestry University, Fuzhou, China; ^3^Texas A&M AgriLife Research Center at Weslaco, Texas A&M University System, Weslaco, TX, United States; ^4^Department of Plant Biology, University of Illinois at Urbana–Champaign, Urbana, IL, United States; ^5^Department of Plant Pathology and Microbiology, Texas A&M University, College Station, TX, United States

**Keywords:** sugarcane, *Saccharum*, polyploidization, genetic divergence, retrotransposon

## Abstract

Sugarcane (*Saccharum* spp. hybrids) is an economically important crop widely grown in tropical and subtropical regions for sugar and ethanol production. However, the large genome size, high ploidy level, interspecific hybridization and aneuploidy make sugarcane one of the most complex genomes and have long hampered genome research in sugarcane. Modern sugarcane cultivars are derived from interspecific hybridization between *S. officinarum* and *S. spontaneum* with 80–90% of the genome from *S. officinarum* and 10–20% of the genome from *S. spontaneum*. We constructed bacterial artificial chromosome (BAC) libraries of *S. officinarum* variety LA Purple (2n = 8x = 80) and *S. spontaneum* haploid clone AP85-441 (2n = 4x = 32), and selected and sequenced 97 BAC clones from the two *Saccharum* BAC libraries. A total of 5,847,280 bp sequence from *S. officinarum* and 5,011,570 bp from *S. spontaneum* were assembled and 749 gene models were annotated in these BACs. A relatively higher gene density and lower repeat content were observed in *S. spontaneum* BACs than in *S. officinarum* BACs. Comparative analysis of syntenic regions revealed a high degree of collinearity in genic regions between *Saccharum* and *Sorghum bicolor* and between *S. officinarum* and *S. spontaneum*. In the syntenic regions, *S. spontaneum* showed expansion relative to *S. officinarum*, and both *S. officinarum* and *S. spontaneum* showed expansion relative to sorghum. Among the 75 full-length LTR retrotransposons identified in the *Saccharum* BACs, none of them are older than 2.6 mys and no full-length LTR elements are shared between *S. officinarum* and *S. spontaneum*. In addition, divergence time estimated using a LTR junction marker and a syntenic gene shared by 3 *S. officinarum* and 1 *S. spontaneum* BACs revealed that the *S. spontaneum* intergenic region was distant to those from the 3 homologous regions in *S. officinarum*. Our results suggested that *S. officinarum* and *S. spontaneum* experienced at least two rounds of independent polyploidization in each lineage after their divergence from a common ancestor.

## Introduction

Sugarcane (*Saccharum* spp. hybrids) produces approximately 80% of the world’s sugar production and is also an important source of biomass. Due to its high productivity, sugarcane is used as biorefineries for the production of biomass, bioenergy and biomaterials (Botha and Moore, 2014; Gómez-Merino et al., 2014). Sugarcane belongs to the genus *Saccharum* that was traditionally divided into six species, two wild species *S. spontaneum* and *S. robustum*, and four cultivated species *S. officinarum, S. edule, S. barberi*, and *S. sinense* (Zhang et al., 2013). However, as originally proposed by Irvine (1999), recent evidence based on morphological, cytological and population structure supported the classification of genus *Saccharum* into two horticultural species, *S. spontaneum* and *S. officinarum*, of which the latter one includes the other four *Saccharum* species and their interspecific hybrids (Zhang et al., 2013). *Saccharum* spp. and *Sorghum bicolor* belong to the grass tribe Andropogoneae in the subfamily Panicoideae. Within the tribe Andropogoneae, *Saccharum, Miscanthus, Erianthus, Narenga*, and *Sclerostachya* form a closely related interspecific breeding group - commonly known as the ‘Saccharum complex.’

*Saccharum officinarum* (2n = 80) has high sugar content and low fiber, but poor disease resistance. *S. spontaneum* (2n = 36–128) is a low sugar, high fiber, disease-resistant species. Modern sugarcane cultivars are mainly derived from interspecific hybridization between *S. officinarum* and *S. spontaneum* to combine high sugar content from *S. officinarum* and disease resistance from *S. spontaneum*. Modern sugarcane hybrids are complex polyploids and aneuploids (2n = 80–140) and are comprised of 70–80% of chromosomes from *S. officinarum*, 10–20% from *S. spontaneum*, and 10% recombinants (D’Hont et al., 1996). The uneven progenitor genome contribution in the interspecific hybrids of sugarcane is due to a phenomenon called female restitution, wherein chromosome transmission is 2n from the female parent *S. officinarum* and n from the male parent *S. spontaneum* (Bremer, 1961).

Whole genome duplication (polyploidy) is common in plants and has been linked to rapid speciation and adaption (Otto and Whitton, 2000; Soltis et al., 2009; Van de Peer et al., 2017). Polyploids are classified as autopolyploids, allopolyploids, or segmental allopolyploids (Stebbins, 1947). Autopolyploids arise via whole genome duplication within the same species; allopolyploids arise via hybridization between two different species with concominant genome doubling; and segmental allopolyploids carry two partially differentiated genomes (Stebbins, 1947). Multiple rounds of ancient (paleo) and/or recent polyploidization events are evident in most angiosperm genomes (Soltis et al., 2009; Jiao and Paterson, 2014). Polyploidization is typically followed by genomic reorganization/fractionation that over time returns the genome to diploid state (Langham et al., 2004; Adams and Wendel, 2005). All the species in the genus *Saccharum* are polyploid and there is no related diploid or tetraploid progenitors known. Despite high ploidy, *Saccharum* species form mainly bivalents at meiosis, and display varying degrees of polysomy and preferential pairing among chromosomes. *S. robustum* shows high proportion of preferential pairing, *S. officinarum* shows some preferential pairing, *S. spontaneum* shows no preferential pairing, and the hybrids of *S. officinarum* and *S. spontaneum* display a continuous range of pairing affinities between chromosomes (D’Hont et al., 2008).

Assumption of molecular clock is useful for estimation of divergence time between species by comparing the divergence between genomic features such as genes and/or TEs. However, many factors contribute to the variation in molecular date estimates including the uncertainty in the absolute age of the evolutionary event used to calibrate the molecular clock, the use of different genes or genomic regions that may be under different selective constraints, and different methods used to estimate divergence times (Gaut et al., 1996; Gaut, 2002). The average synonymous substitution rate obtained from the grass adh1/2 alleles (6.5 × 10^-9^ per site per year) estimated by assuming the maize–rice divergence time of 50 million years (mys) (Gaut et al., 1996) is commonly employed to estimate the divergence time in grasses. And, a two-fold higher substitution rate of 1.3 × 10^-8^ mutations per site per year is commonly used to estimate the insertion time of LTR retrotransposons (Ma and Bennetzen, 2004).

The polyploidization and divergence history of *Saccharum* lineage remains poorly understood. The octaploid sugarcane genome has experienced two rounds of whole genome duplication since its divergence from sorghum, and is thus, an ideal system to study the impact of polyploidy on speciation, subgenome divergence and genomic adaption to the duplicated state (Kim et al., 2014). Recent studies have variably estimated the divergence time of sugarcane and sorghum (Jannoo et al., 2007; Wang et al., 2010; Kim et al., 2014; Vilela et al., 2017) and different models have been proposed for the type and time of polyploidy in sugarcane (Kim et al., 2014; Vilela et al., 2017). Kim et al. (2014) proposed that an allopolyploidy in the common ancestor of *Miscanthus*-*Saccharum* resulted in the divergence of Saccharinae and Sorghinae subtribes, and subsequent *Saccharum*-specific autopolyploidy resulted in random chromosome pairing within a group but infrequent pairing between groups. Although this scenario explains preferential pairing observed in *S. officinarum*, it does not explain no preferential pairing in *S. spontaneum*. Vilela et al. (2017) suggested that *S. officinarum* and *S. spontaneum* lineages each experienced independent autopolyploidization after their divergence. Further research is still needed to fully understand the polyploidization and divergence history of sugarcane.

The large genome size, high ploidy level, interspecific hybridization and aneuploidy make sugarcane one of the most complex genomes and have long hampered genome research in sugarcane. The two sugarcane progenitors, *S. officinarum* and *S. spontaneum* are an ideal genomic resource to infer evolutionary history of the genus *Saccharum*, as well as to study the complex mechanisms leading to the superior productivity of sugarcane cultivars. In this study, we selected and sequenced homo/homeologous BACs from *S. officinarum* and *S. spontaneum* BAC libraries, and conducted comparative analysis to assess variation in genome size, and mode and time of divergence between *Saccharum* and sorghum, and between the modern sugarcane progenitor species, *S. spontaneum* and *S. officinarum*.

## Materials and Methods

### Construction of *Saccharum officinarum* and *Saccharum spontaneum* BAC Libraries

Young leaf tissue was harvested from *Saccharum officinarum* variety LA Purple (2n = 8X = 80) and *S. spontaneum* haploid clone AP85–441 (2n = 4X = 32) and used for nuclei extraction. Nuclei was isolated following the protocol described by Ming et al. (2001). The high molecular weight DNA was extracted from nuclei and then embedded in agarose and partially digested with *Hin*d III. The fraction at approximately 120 kb was recovered and cloned into *Hin*d III linearized pSMART BAC vector (Lucigen)^1^. A total of 76,800 colonies for LA Purple and 38,400 colonies for AP85-441 were archived in 384-well plates with freezing medium. BAC clones were spotted onto high-density nylon filters (Performa II Nylon Filters, Genetix) using Q-Pix2 (Genetix) for hybridization screening.

### Screening the BAC Libraries

PCR primers targeting the genes involved in sucrose, lignin, and cellulose biosynthesis pathways were designed using Primer Premier 5 software^2^ and used for RT-PCR amplification. PCR products were purified using Wizard^®^ SV Gel and PCR Clean-Up System (Promega) and used as probes to screen the BAC libraries. Hybridization screening of the BAC libraries was performed using the method described by Yu et al. (2011). High-density membranes of the BAC libraries were prehybridized in 0.5 M Na_2_HPO_4_, 7% SDS, 1 mM EDTA, 100 μg ml^-1^ heat-denatured herring sperm DNA for at least 4 h. Probes were labeled using a random primer labeling system (NEBlot Kit, New England Biolabs). The hybridization was performed overnight at 55°C in 0.5 M Na_2_HPO_4_, 7% SDS, 1 mM EDTA, 100 μg ml^-1^ heat-denatured herring sperm DNA with ^32^P-labeled probes. Hybridized membranes were washed twice in 0.5 × SSPE/0.5% SDS for 10 min each time.

### Verification of BAC Clones

BAC DNA was isolated using the alkaline lysis method and digested with *Hin*d III. The digested DNA samples were electrophoresed through a 0.8% agarose gel. After electrophoresis, the gel was blotted onto Amersham Hybond N+ membranes (GE Healthcare) using standard methods (Sambrook et al., 1987). Southern hybridization was performed using the method described by Yu et al. (2011).

### Sequencing BAC Clones and Sequence Assembly

BAC DNA was extracted from selected BAC clones using QIAGEN Large-Construct kit (Qiagen) and used for pyrosequencing on a Roche 454 GS FLX+ Titanium platform at Texas A&M AgriLife Genomics & Bioinformatics Service. Each BAC clone was labeled with a unique multiplex identifier and every 12 BACs were pooled at equal amount and sequenced on one region of a four-gasket sequencing run.

The sequence reads were assembled using Newbler with default parameter settings. Sequence reads matching the *Escherichia coli* genome and the BAC vector were removed and trimmed. The sequence gaps were filled by primer walking and/or directly sequencing PCR products when possible.

### Sugarcane Repeat Database and Estimation of Repeat Content

We used both de novo and structure-based approaches to identify high-copy number repeats in the 475 sugarcane BACs, including the BACs assembled in this study and 378 sugarcane BACs downloaded from GenBank. The BACs downloaded from GenBank included 2 BACs of AP85-441 (*S. spontaneum*), 4 BACs of LA Purple (*S. officinarum*), and 372 BACs of the modern sugarcane cultivar R570 (an interspecific hybrid between *S. officinarum* and *S. spontaneum*) (**Supplementary Table S1**). The TEdenovo pipeline from the REPET package (Flutre et al., 2011) and RepeatModeler (Smit and Hubley, 2008) were used to *de novo* predict sugarcane repeats by an all-by-all comparison with default parameters. Among the de novo identified repeats that were classified as chimeric or SSR by the TEdenovo, those with less than 10 copies (at 80% coverage threshold) in the sugarcane BACs and those with matches to repeat-masked plant CDS sequences were filtered. Finally, we used ProtExcluder^3^ to remove protein coding genes from repeat library by mapping putative repeats against the plant protein database where transposon proteins were excluded^4^. In addition, LTR_finder (Xu and Wang, 2007) was used to predict full-length LTR retrotransposon and TRIMs. MITE_hunter (Han and Wessler, 2010) was used to generate consensus representative sequences for sugarcane MITEs. All repeats were combined and clustered using VSEARCH (Rognes et al., 2016). The consensus sequences obtained from VSEARCH were then annotated using the RepeatClassifier script of the RepeatModeler package by comparison to the Repbase database (Jurka et al., 2005). The final non-redundant repeat database was made using CD-Hit-EST (Li and Godzik, 2006) at 80% sequence identity. The full-length LTR representatives were classified by comparing their RT domains to the ones of the classified sugarcane LTR retrotransposons (Domingues et al., 2012) and to the Gypsy Database 2.0 (Llorens et al., 2011). The repeat content of the *Saccharum* BACs was estimated by RepeatMasker (Smit et al., 1996) using the custom sugarcane repeat database.

### Gene Model Prediction and Annotation

We used MAKER (Cantarel et al., 2008) to annotate genes in the assembled *Saccharum* BACs. The gene models were predicted based on the combined available evidence based on matches to the repeat database, EST/cDNA, and proteins, as well as predictions by *ab initio* gene prediction programs. The repeats database included the MIPS Repeat Element Database (mips-REdat)^5^ (Nussbaumer et al., 2013), the Repbase repeat database^6^ (Jurka et al., 2005) and the sugarcane repeats identified in this study. The transcript evidence included five RNAseq assemblies and the in-house sugarcane ESTs. The protein evidence included the plant protein database from the ProtExcluder package and plant proteins downloaded from Phytozome (Goodstein et al., 2012). Gene predictors, SNAP (Korf, 2004) using *O. sativa* hmm parameter and AUGUSTUS (Stanke et al., 2006) using maize hmm parameter, were run within MAKER on both masked and unmasked sequence and gene models with the best AED score per locus was selected. Gene models with evidence support (AED score > 1) or PFAM domains with default parameters in InterProScan were selected. The gene models were then annotated based on homology to the UniRef90 protein database (Suzek et al., 2007).

### Estimation of Insertion Time of Full Length LTR Retrotransposon Elements

The full-length LTR retrotransposons were identified based on full-length matches to the LTR consensus sequences using BLAST. The pairwise alignment between 5′ and 3′ LTR of each copy was generated by BLAST2seq. Pairwise alignments were conducted to estimate the number of base substitutions per site based on the Kimura 2-parameter model using MEGA7 (Kumar et al., 2016). The divergence time was estimated using the mutation rate of 1.3 × 10^-8^ mutations per site per year (Ma and Bennetzen, 2004). We used junctions formed at the LTR insertion sites as markers (Luce et al., 2006) to identify shared insertion sites between and within *S. officinarum* and *S. spontaneum*. Up to 2 kb of the shared TE sequence (smaller than 2 kb in case of truncation) at the junction site was used for estimation of sequence divergence between paired BACs using the mutation rate of 1.3 × 10^-8^ mutations per site per year (Ma and Bennetzen, 2004).

### Identification of Syntenic Gene Pairs and Calculation of the *Ka*/*Ks* Values

The BAC sequences were uploaded to COGE. SynMap2 at CoGe (Lyons and Freeling, 2008) was used to identify syntenic gene pairs between sorghum and *Saccharum* species (*S. officinarum* and *S. spontaneum*), and between *S. officinarum* and *S. spontaneum*. The homologous gene pairs were identified using discontinuous MegaBLAST algorithm and *e*-value less than 0.001. Relative gene order was used to compute chains of syntenic genes using DAGchainer (Haas et al., 2004), allowing a maximum distance of 30 genes and minimum number of 2 aligned gene pairs. A coverage depth ratio of 1 sorghum to 8 sugarcane genes was used. The pairwise CDS alignments for the syntenic gene pairs were generated using MACSE (Ranwez et al., 2011), and the rate of synonymous (*Ks*) and non-synonymous (*Ka*) substitutions for each syntenic gene pair was calculated using the Nei–Gojobori model in MEGA 7.0 (Kumar et al., 2016). The *Ks* values were converted to divergence times using the average synonymous substitution rate of the grass adh1/2 alleles (6.5 × 10^-9^ per site per year) estimated by assuming the maize–rice divergence time of 50 mys (Gaut et al., 1996).

### Visualization of Orthologous BACs

The orthologous BACs were visualized using EasyFig (Sullivan et al., 2011). The repeat regions were lower case masked to allow BLAST extension from genes into neighboring shared ancestral repeats and suppress cross matches between other repeat regions.

## Results

### BAC Library Construction, and Selection and Sequencing BACs

A BAC library of AP85-441 (*S. spontaneum*, 2n = 4X = 32) and a BAC library of LA Purple (*S. officinarum*, 2n = 8X = 80) were constructed using *Hin*d III partially digested high-molecular-weight DNA. The BAC library of AP85-441 consists of 38,400 clones and the BAC library of LA Purple consists of 76,800 clones. We randomly picked 120 clones from each library to estimate the average insert size. The average insert size of the BAC library of AP85–441 was estimated at 110 kb and the one of the BAC library of LA Purple was estimated at 120 kb. Since the genome sizes of AP85–441 and LA Purple are 3.36 Gb/2C and 7.66 Gb/2C (Zhang et al., 2012), the BAC libraries of AP85-441 and LA Purple represent approximately 1.26 and 1.20 genome equivalents, respectively.

We used the probes designed for the genes on sucrose, lignin, and cellulose biosynthesis pathways to screen the two *Saccharum* BAC libraries and selected 53 LA Purple BACs (named with So) and 44 AP85–441 BACs (named with Ss) for sequencing. The total length of the assembled sequence for the 97 BACs is 10,858,850 bp, 5,847,280 bp for the 53 So BACs and 5,011,570 bp for the 43 Ss BACs. These sequences represent approximately 0.08% of the LA purple genome and 0.15% of the AP85–441 genome based on an estimated genome size of 7.66 Gb for LA Purple and 3.36 Gb for AP85–441 (Zhang et al., 2012).

Among the 97 BACs, 79 BACs (41 So BACs and 38 Ss BACs) could be completed by primer walking, and each was assembled into a single contig. Seven BACs (5 So BACs and 2 Ss BACs) were each assembled into two ordered and oriented contigs. Three BACs (2 So BACs and 1 Ss BACs) were each assembled into three ordered but not oriented contigs. The rest 8 BACs (5 So BACs and 3 Ss BACs) were assembled into 7–21 contigs, of which the internal contigs couldn’t be ordered and oriented. Sequence assembly statistics of the 97 BACs was summarized in **Supplementary Table S2**. The assembled BACs have been deposited in GenBank and the GenBank accession numbers are MH182499-MH182581 and KU685404-KU685417.

### Gene Prediction and Annotation

We used MAKER to annotate the *Saccharum* BACs and obtained 778 gene models that had an Annotation Edit distance (AED) score < 1.00 and/or had a PFAM domain. The AED score measures the congruence between an annotation with its supporting evidence, and ranges from 0 to 1, where value 0 indicates perfect match of annotation to the evidence and value 1 indicates no evidence support of annotation. We filtered 29 gene models that had TE-related PFAM domains and AED value of 1.00. The remaining 749 genes models (401 from Ss BACs and 348 from So BACs) had AED score < 1.00 and/or had a non-TE related PFAM domain. The Ss BACs have a relatively higher gene density (approximately 80 genes per Mb) compared to the So BACs (63 genes per Mb), which is consistent with the lower repeat content in Ss BACs than in So BACs (See details in “Repeat content in selected *Saccharum* BACs” and **Table 1**). The functional annotation of gene models was based on sequence similarity search in the UniRef90 database (**Supplementary Table S3**).

**Table 1:** Summary of repeat content of *Saccharum officinarum* and *Saccharum spontaneum* BACs.

Element	*S. officinarum* BACs		*S. spontaneum* BACs	
	
	5,848,270 bp		5,012,466 bp	
	Masked (bp)	Masked (%)	Masked (bp)	Masked (%)
**Interspersed repeats**				
DNA transposons				
Unknown	2865	0.05	7135	0.14
MULE-MuDR	45154	0.77	56697	1.13
PIF-Harbinger	109620	1.87	115483	2.30
TcMar-Stowaway	42664	0.73	55349	1.10
CMC-EnSpm	32453	0.55	34989	0.70
hAT (unclassified)	3085	0.05	2834	0.06
hAT-Ac	16941	0.29	8824	0.18
hAT-Tag1	1149	0.02	6434	0.13
hAT-Tip100	6316	0.11	3330	0.07
Helitron	1958	0.03	3119	0.06
**Retroelements**				
LTRs				
Unknown	3801	0.06	5160	0.10
Copia (unclassified)	39635	0.68	18973	0.38
Copia-Ale	51928	0.89	90752	1.81
Copia-Ang	109938	1.88	80897	1.61
Copia-Iva	23128	0.40	22273	0.44
Copia-Max	754158	12.90	451646	9.01
Copia-Tor	34550	0.59	21755	0.43
Gypsy (unclassified)	**346401**	**5.92**	**142809**	**2.85**
Gypsy-Ath	63989	1.09	117340	2.34
Gypsy-Crm	42722	0.73	21850	0.44
Gypsy-Del	**816169**	**13.96**	**366879**	**7.32**
Gypsy-Rei	43787	0.75	29932	0.60
Gypsy-Tat	292738	5.01	278733	5.56
LINE/L1	10443	0.18	8146	0.16
LINE/RTE-BovB	35478	0.61	12619	0.25
SINE/tRNA	861	0.01	1292	0.03
Unknown	45898	0.78	42075	0.84
**Total interspersed repeats**	**2977829**	**50.92**	**2007325**	**40.05**
Simple sequence repeats				
Low complexity	7814	0.13	7660	0.15
Satellite	10594	0.18	12551	0.25
Simple repeat	109561	1.87	47434	0.95
**Total masked**	**3105798**	**53.11**	**2074970**	**41.40**

*Bold values mark large differences in repeat content between the two sugarcane progenitors.*

Approximately 86% of the gene models in Ss BACs and 89% of the gene models in So BACs had an AED ≤ 0.5 (**Supplementary Figure S1**). Although six gene models were annotated as TE-related genes, we did not filter them because they could be bona fide expressed TEs as evidenced by their AED scores < 1.00. Thirty-two gene models may be pseudogenes because they had an AED score of 1.00 but contained non-TE related PFAM domains. Twenty-eight gene models with AED < 1.00 might be caused by artifacts or spurious protein alignments as they do not contain a PFAM domain and had an eAED score of 1.00.

### Repeat Content in Selected *Saccharum* BACs

We compiled a custom repeat database for sugarcane and used RepeatMasker to estimate the repeat content in selected *Saccharum* BACs using the sugarcane repeat library. The So BACs and Ss BACs contain 53 and 41% repetitive sequences, respectively (**Table 1**). This repeat content may be underestimated because some bona fide repeats may escape detection due to their low copy number in the examined BACs and the repeat consensus sequences may not capture the full range of the repeat sequence variation. Like in other plants, LTR retrotransposons are the most abundant repeat in *Saccharum* BACs, accounting for 45% of the So BAC sequences and 33% of the Ss BAC sequences. The maximus lineage of the Ty1/Copia type and the Del lineage of the Ty3/Gypsy type elements form the largest fraction of LTR retrotransposon in both So and Ss BACs. In general, So BACs contain a higher total interspersed repeat content and total LTR retrotransposon content than Ss BACs. For the major LTR retrotransposons, a much higher percentage of Max lineage (Copia), Del lineage (Gypsy), and unclassified Gypsy LTR retrotransposons was observed in So BACs than in Ss BACs. Some of the unclassified Gypsy elements are possibly LARD elements that are related to Del.

### Identification of Syntenic Regions Between *Saccharum* and Sorghum and Between *S. officinarum* and *S. spontaneum*

**FIGURE 1:**
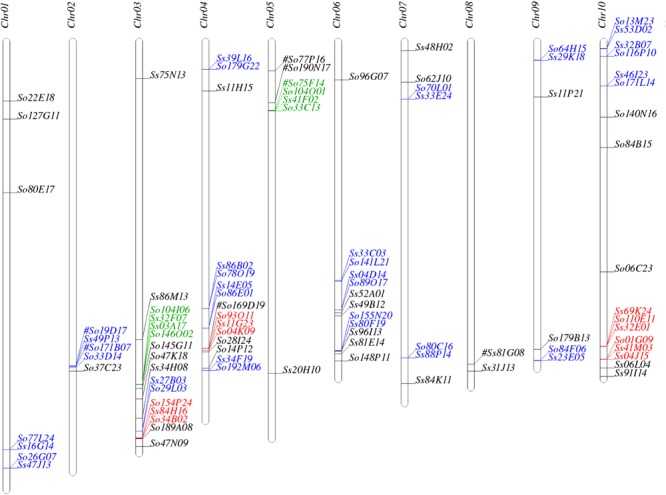
Mapping the *Saccharum* BACs on sorghum chromosomes. Homologous groups of two, three, and four BACs are shown in blue, red, and green, respectively. ^#^: These homologous BACs were identified by Blast.

We used SynMap to identify syntenic regions between *Saccharum* and sorghum genomes. Fifty-seven syntenic blocks were identified by mapping 87 *Saccharum* BACs (45 So and 42 Ss BACs) against sorghum genome based on synteny of 205 So and 227 Ss gene models to the sorghum gene models (**Supplementary Table S3**). The syntenic regions for seven *Saccharum* BACs (6 So BACs and 1 Ss BAC) could not be identified by SynMap due to lack of a minimum of two genes syntenic to sorghum genes. We individually BLASTed these 7 *Saccharum* BACs into sorghum genome and identified seven syntenic blocks of which three have been identified by other *Saccharum* BACs using SynMap. The map location of the 94 *Saccharum* BACs on sorghum chromosomes are summarized in **Figure 1** and **Supplementary Table S3**. Based on the map location in sorghum genome, we grouped the 94 *Saccharum* BACs into 61 homology groups. We further grouped the 61 homology groups into 8 types based on the number of So and Ss BACs mapped to a sorghum syntenic region. The eight types of homology groups were named Sb-2So-2Ss, Sb-3So-1Ss, Sb-2So-0Ss, Sb-2So-1Ss, Sb-1So-2Ss, Sb-0So-1Ss, Sb-1So-1Ss, Sb-1So-0Ss. The detailed information of the 61 homology groups can be found in **Supplementary Table S4**.

**FIGURE 2:**
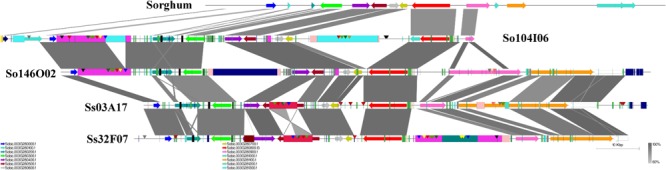
A schematic representation of a syntenic region between *Saccharum* and sorghum and between *S. officinarum* and *S. spontaneum*. The color-coded arrows represent genes, rectangles represent repeats, and conserved domains in transposable elements are represented by pointers. The blast similarity between annotated genic regions is shown by connectors in gray color gradient. A high degree of co-linearity is shared between *Saccharum* and sorghum and between *S. officinarum* and *S. spontaneum*. The large TEs are shared by homologous regions within the same species but not by the ones from different *Saccharum* species.

**FIGURE 3:**
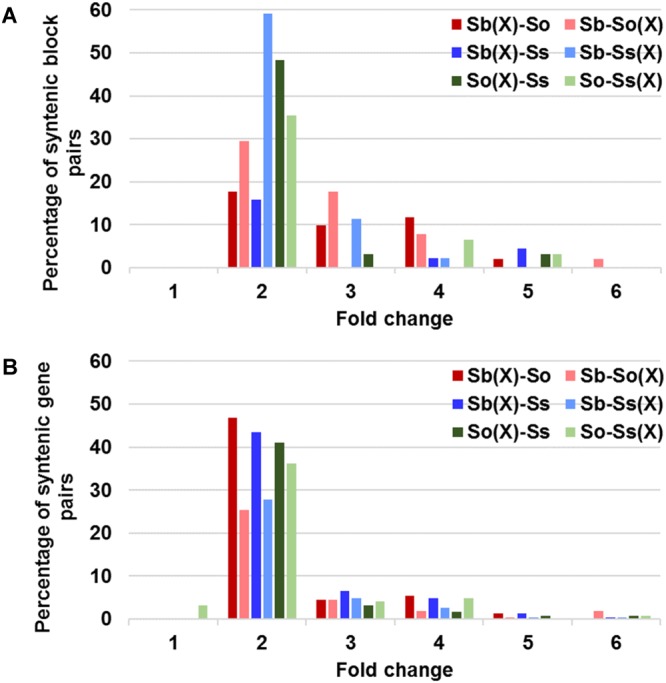
Relative size expansion between *Saccharum* and sorghum and between *Saccharum officinarum* and *Saccharum spontaneum* in syntenic blocks **(A)** and syntenic gene pairs **(B)**. X: expansion.

A schematic representation of a syntenic region between sorghum, *S. officinarum* (BACs So104I06 and So146O02), and *S. spontaneum* (BACs Ss03A17 and Ss32F07) is shown in **Figure 2**. The schematic for additional homologous groups is shown in **Supplementary Figure S2**. A high degree of collinearity in genic regions was observed between *Saccharum* and sorghum and between *S. officinarum* and *S. spontaneum*. The collinearity was interrupted by interspersed repeats (**Figure 2**). We used the mRNA coordinates of the syntenic genes to delineate and assess the pairwise difference in the length of the syntenic regions and the syntenic genes from sorghum and *Saccharum*. Of the 51 syntenic regions identified between *S. officinarum* and sorghum genomes, 29 showed expansion in *S. officinarum* and 22 showed expansion in sorghum (**Figure 3A**). Of the 44 syntenic regions identified between *S. spontaneum* and sorghum gnomes, 33 showed expansion in *S. spontaneum* and 11 showed expansion in sorghum (**Figure 3A**). And, of the 31 syntenic regions identified between *S. officinarum* and *S. spontaneum*, 17 had expanded in *S. officinarum* and 14 had expanded in *S. spontaneum*. Most expanded regions had up to 2-fold expansion, although there were few outliers (>3-fold expansion) that might be caused by genome rearrangements, genome mis-assembly and/or high repeat insertions. Including the outliers, the total length of the syntenic regions in sorghum was 1.1-fold of *S. officinarum* and 0.96-fold of *S. spontaneum*. After excluding the outliers (with >3-fold expansion), the total length of syntenic regions in sorghum was 0.92-fold of *S. officinarum* and 0.77-fold of *S. spontaneum*. Overall, *S. spontaneum* showed expansion relative to *S. officinarum*, and both *S. officinarum* and *S. spontaneum* showed expansion relative to sorghum.

We also compared the expansion within the annotated genes and found that the expansion in genic regions was at a much smaller scale (**Figure 3B**). Approximately half or more than half (46–65%) of gene pairs showed < 1.3-fold expansion. Approximately 20% of *S. officinarum* and *S. spontaneum* genes showed < 1.3-fold expansion relative to sorghum genes, approximately 27% of sorghum genes showed < 1.3-fold expansion relative to *S. officinarum* and *S. spontaneum* genes, and approximately 35% of *S. officinarum* genes and 30% of *S. spontaneum* genes had <1.3-fold expansion relative to *S. spontaneum* and *S. officinarum* genes, respectively. Our result indicated that the expansion of syntenic regions in *Saccharum* was largely caused by the expansion in the intergenic regions.

### Evolutionary Divergence Between Syntenic Gene Pairs

**FIGURE 4:**
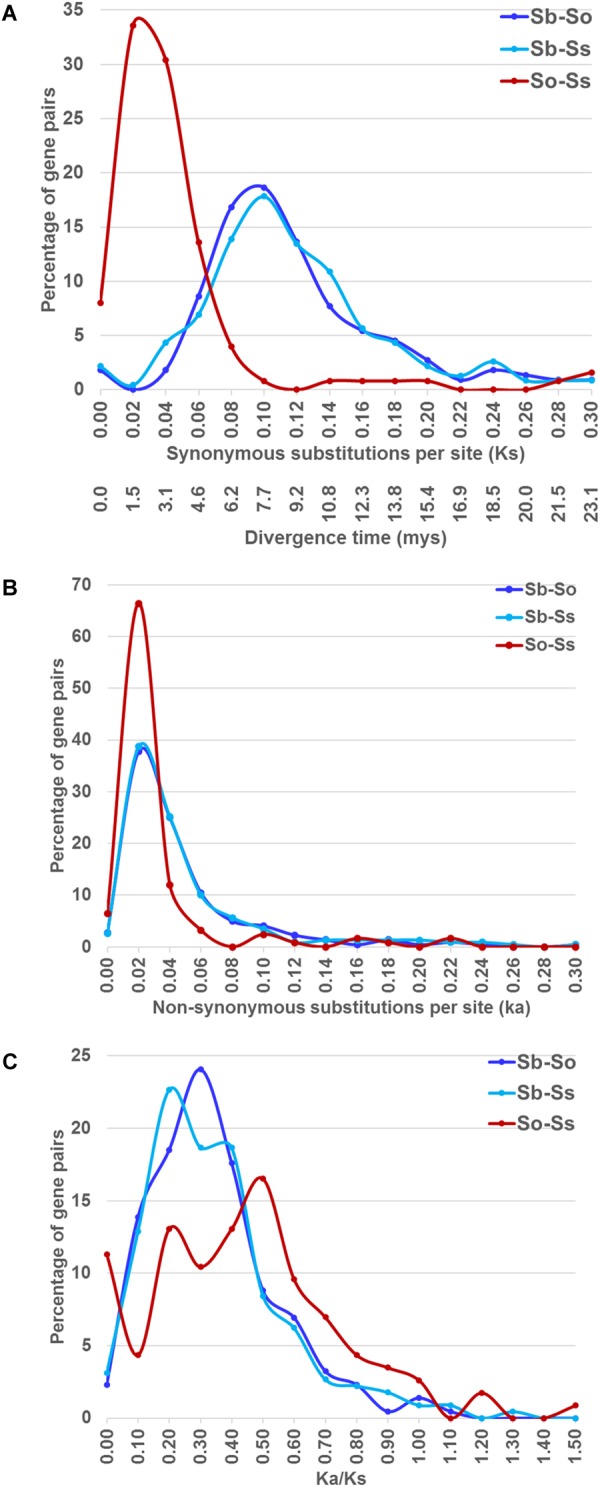
The frequency distribution of the *Ks* and the corresponding divergence times **(A)**, *Ka* **(B)**, and *Ka*/*Ks* values **(C)** between sorghum and *Saccharum*, and between *S. officinarum* and *S. spontaneum*.

We estimated the *Ks* and *Ka* values of syntenic gene pairs between sorghum and *S. officinarum*, sorghum and *S. spontaneum*, and *S. officinarum* and *S. spontaneum*. The frequency distribution of the *Ks, Ka*, and *Ka*/*Ks* for the three comparisons is shown in **Figure 4**. The distribution of the *Ks* and *Ka* values of sorghum/*S. officinarum* and sorghum/*S. spontaneum* showed similar patterns. The peak *Ks* value for syntenic genes between sorghum and *S. officinarum* and between sorghum and *S. spontaneum* was 0.10 and the estimated divergence time was 7.7 mys. The peak *Ks* value of syntenic gene pairs between *S. officinarum* and *S. spontaneum* was 0.02 and the estimated divergence time was 1.5 mys. The peak *Ka* value for syntenic gene pairs was 0.2 for sorghum/*S. officinarum* and sorghum/*S. spontaneum*, and 0.1 for *S. officinarum*/*S. spontaneum*. The *Ka*/*Ks* values of most gene pairs (86–98%) was less than 1.00 suggesting that most syntenic gene pairs are under purifying selection (**Table 2**).

**Table 2:** Number of syntenic gene pairs used for calculation of *Ks, Ka*, and *Ka*/*Ks* ratios.

	Sb-So	Sb-Ss	So-Ss
**Total gene pairs**	**220**	**230**	**125**
Pairs with *Ks* < 0.5	208 (94.55%)	219 (95.22%)	122 (97.60%)
Pairs with *Ka* < 0.5	209 (95.00%)	223 (96.96%)	120 (96.00%)
Pairs with *Ka*/*Ks* < 1.00^∗^	215 (97.73%)	221 (96.09%)	107 (85.60%)

*^∗^The *Ka*/*Ks* value for 4 Sb–So (1.82%), 5 Sb–Ss (2.17%), and 10 So–Ss (7.87%) gene pairs could not be determined because the *Ks* values of these comparison were 0.*

### Insertion Time of LTR Retrotransposon Lineages in *S. officinarum* and *S. spontaneum*

**FIGURE 5:**
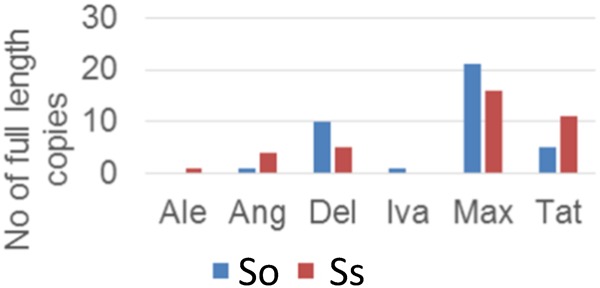
Full-length LTR retrotransposon copies in So and Ss BACs.

**FIGURE 6:**
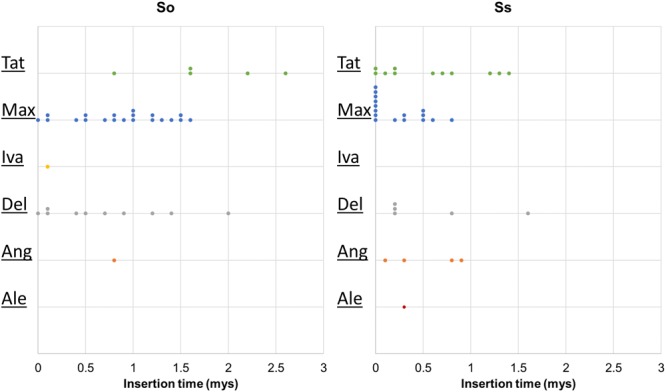
Insertion time of LTR retrotransposon families. The insertion time of LTR retrotransposon families in So (left graph) and Ss (right graph) BACs are shown. The *X* axis represents the insertion time (mys). Each dot in the graph represents insertion time of one element and these are stacked when more than one element has the same insertion time for easy visualization of copy number. The insertion time was calculated based on substitution rate of 1.3 × 10^-8^ (Ma and Bennetzen, 2004).

Retrotransposon activation can be triggered by many factors including genome duplication. Therefore, it would be interesting to see the impact of genome duplication on LTR retrotransposons in *Saccharum* genomes. We extracted the full-length LTR retrotransposon copies from the So and Ss BACs and estimated their insertion times. The number of full-length LTR retrotransposon copies extracted from So (38 copies) and Ss (37 copies) BACs were similar (**Figure 5**). However, there were more Del and Max lineage members in So BACs than in Ss BACs. Overall, the full-length LTR retrotransposons in Ss BACs are younger than in So BACs (**Figure 6**). In Ss BACs, 67 and 89% of the full-length LTR retrotransposons are younger than 0.5 and 1 million years, respectively. In So BACs, 32 and 60% of the full-length elements are younger than 0.5 and 1 million years, respectively (**Figure 6**). None of the full-length LTR retrotransposons in Ss BACs are older than 2 mys, which is the estimated time when *S. officinarum* and *S. spontaneum* diverged. Interesting, none of the intact LTR retrotransposons were shared between *S. officinarum* and *S. spontaneum*.

**FIGURE 7:**
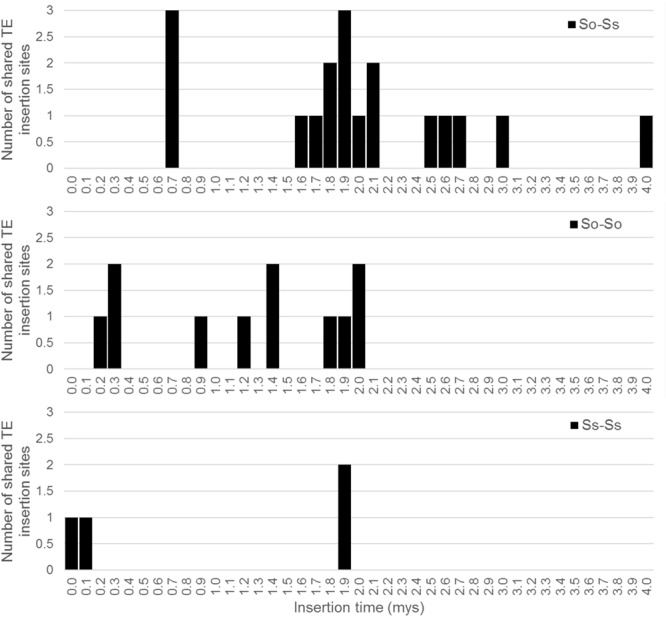
Insertion times of LTR junction markers shared between *Saccharum* BACs. The *X* axis represents the insertion time (mys) and the *Y* axis represents the number of shared LTR junction markers. The insertion time was calculated based on substitution rate of 1.3 × 10^-8^ per site per year (Ma and Bennetzen, 2004).

Since *S. officinarum* and *S. spontaneum* diverged from a common ancestor recently, we would expect that remnants of some LTR retrotransposon fragments predating the divergence of *S. officinarum* and *S. spontaneum* have been retained and can be identified in the two genomes. TE insertions into the genome or within other TEs form unique junctions at their insertion sites, which can be used as markers even though the original copy has mostly degenerated (Luce et al., 2006). We identified signatures of shared LTR retrotransposon insertions between paired homologous BACs. A total of 18 LTR junction markers were identified in paired homologous BACs between *S. officinarum* and *S. spontaneum*, 11 were identified in paired homologous BACs within *S. officinarum*, and 4 were identified in paired homologous BACs within *S. spontaneum* (**Table 3**). It was estimated that *S. officinarum* and *S. spontaneum* diverged from a common ancestor approximately 1.5 – 2 mys (Jannoo et al., 2007). Interestingly, the insertion times of all the LTR junction markers shared by homologous BACs within *S. officinarum* and within *S. spontaneum* were estimated at ≤2 mys, while the insertion times of all except three LTR junction markers shared between *S. officinarum* and *S. spontaneum* were estimated > 1.5 mys (**Figure 7**).

**Table 3:** LTR junction marker identified in paired homologous BACs in *Saccharum* BACs.

Marker name	BAC1 ID	BAC1 coordinate		BAC2 ID	BAC2 coordinate		Marker type	Aligned length (%)	Identity (%)
So/Ss.1	So70L01	105063	105165	Ss33E24	101220	101315	End_Del	95.556	45
So/Ss.2	So01G09	48178	48075	Ss04J15	99569	99672	End_Max	98.333	60
So/Ss.3	So01G09	48178	48075	Ss41M03	9167	9065	End_Max	98.333	60
So/Ss.4	So104O01	49103	49198	Ss41F02	20444	20539	End_Max	100	60
So/Ss.5	So141L21	69996	70095	Ss33C03	63566	63664	End_Max	100	60
So/Ss.6	So141L21	92850	92947	Ss33C03	80868	80964	End_Max	98.333	60
So/Ss.7	So192M06	21741	21644	Ss34F19	35289	35386	End_Max	100	60
So/Ss.8	So33C13	29101	29006	Ss41F02	20444	20539	End_Max	100	60
So/Ss.9	So34B02	58358	58265	Ss84H16	86435	86527	End_Max	93.333	60
So/Ss.10	So75F14	25627	25722	Ss41F02	20444	20539	End_Max	100	60
So/Ss.11	So34B02	99058	99158	Ss84H16	78551	78450	Start_Ang	96.667	60
So/Ss.12	So01G09	50075	49976	Ss41M03	10993	10895	Start_Max	98.333	60
So/Ss.13	So01G09	50075	49976	Ss04J15	97667	97766	Start_Max	96.667	60
So/Ss.14	So141L21	89269	89368	Ss33C03	77272	77364	Start_Max	96.667	60
So/Ss.15	So141L21	68024	68123	Ss33C03	52616	52715	Start_Max	100	60
So/Ss.16	So155N20	60505	60592	Ss80F19	4670	4583	Start_Max	98.333	60
So/Ss.17	So34B02	57372	57471	Ss84H16	87420	87334	Start_Max	96.667	60
So/Ss.18	So86E01	40124	40222	Ss14E05	38389	38474	Start_Max	91.667	60
Ss/Ss.1	Ss04J15	99569	99672	Ss41M03	9167	9065	End_Max	96.667	60
Ss/Ss.2	Ss32E01	33882	33785	Ss69K24	2745	2842	Start_Ale	100	60
Ss/Ss.3	Ss04J15	97667	97766	Ss41M03	10993	10895	Start_Max	98.333	60
Ss/Ss.4	Ss32E01	7753	7654	Ss69K24	28879	28978	Start_Max	100	60
So/So.1	So04K09	13971	14067	So93O11	100606	100510	End_Del	98.276	58
So/So.2	So04K09	45961	46057	So93O11	92738	92643	End_Max	98.333	60
So/So.3	So04K09	45158	45257	So93O11	93534	93435	End_Max	100	60
So/So.4	So104O01	49103	49198	So75F14	25627	25722	End_Max	100	60
So/So.5	So104O01	49103	49198	So33C13	29101	29006	End_Max	100	60
So/So.6	So33C13	29101	29006	So75F14	25627	25722	End_Max	100	60
So/So.7	So104I06	97112	97211	So146O02	3957	3858	End_Tat	98.333	60
So/So.8	So171B07	22361	22461	So33D14	58463	58362	Start_Del	96.667	60
So/So.9	So04K09	46549	46461	So93O11	92160	92248	Start_Max	98.333	60
So/So.10	So04K09	43263	43361	So93O11	95353	95254	Start_Max	98.333	60
So/So.11	So104I06	79770	79868	So146O02	16385	16286	Start_Tat	98.333	60

**FIGURE 8:**
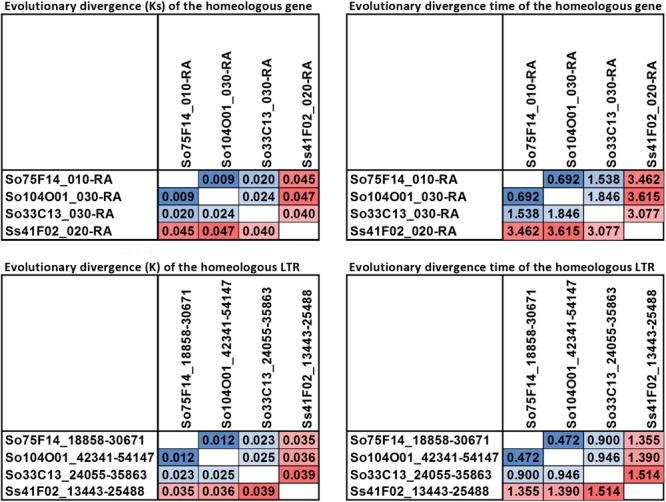
Pairwise evolutionary distance for a homeologous gene and a LTR. The homeologous gene and LTR sequence share a higher similarity among the three So BACs than to the Ss BAC.

The LTR junction marker with the lowest divergence between *S. officinarum* and *S. spontaneum* was present in three So BACs (So104O01, So33C13, and So75F14) and one Ss (Ss41F02) BAC. A 7 kb-long multiple alignment was generated from the homologous region containing the LTR junction marker from the four BACs and used to estimate the divergence time of the intergenic region. The *K* values based on the homologous intergenic region showed that Ss41F02 diverged from the common ancestor of So104O01, So33C13, and So75F14 (*K* = 0.035–0.039) first, followed by the divergence of So33C13 from the common ancestor of So75F14 and So104O01 (*K* = 0.023 and 0.025), and So104O01 and So75F14 diverged the most recently (*K* = 0.012). The same pattern of divergence was observed using the divergence (Ks) of a syntenic gene shared by all four BACs (**Figure 8**).

## Discussion

Sugarcane (*Saccharum*) is closely related to sorghum (*Sorghum bicolor*). The two progenitors of modern sugarcane, *S. officinarum* and *S. spontaneum*, are octoploids, which have experienced two rounds of whole genome duplications since the divergence of *Saccharum* and sorghum. The divergence time between sorghum and sugarcane has been variously estimated at 8–9 mys based on Adh1 gene (Jannoo et al., 2007), 7.7 mys based on 67 pairs of orthologous genes (Wang et al., 2010), 5.0–7.4 mys based on three homologous regions (Vilela et al., 2017), and 5.4 mys by Kim et al. (2014). Similarly, the divergence time of *S. officinarum* and *S. spontaneum* was also variably estimated at 1.5–2 mys based on Adh1 gene (Jannoo et al., 2007), and 2.5–2.8 mys based on TOR haplotypes (Vilela et al., 2017). We estimated the divergence time of sugarcane and sorghum at 7.7 mys (*K*s = 0.10) and the divergence time of *S. officinarum* and *S. spontaneum* at 1.5 mys (*K*s = 0.02) based on synonymous distance between syntenic gene pairs from *S. officinarum, S. spontaneum* and sorghum genomes. Our divergence time estimates overlap with those reported in previous studies and are expected to be more accurate because we used the mutation rate of a much larger number of genes from the two sugarcane progenitors.

The evolutionary history of polyploidization events in the genus *Saccharum* is still debated. Kim et al. (2014) proposed that allopolyploidy occurred in the common ancestor of *Saccharum* and *Miscanthus*, followed by *Saccharum*-specific autopolyploidy based on the distribution of *Ks* value peaks between *Saccharum* and *Miscanthus* paralogs. The authors used sorghum exons to identify paralogous *Miscanthus* exons, which were subsequently used to identify sugarcane paralogs from NCBI EST database. The authors used 2368 pairs of *Miscanthus* exons (equivalent to ∼391 genes, assuming 6.05 exons per transcript estimated for sorghum) to identify sugarcane paralogs from EST database. However, it is not clear whether the sugarcane paralogs were from *S. officinarum* only, as most ESTs in GenBank are from the sugarcane hybrid R570 which contains about 20% of the genome from *S. spontaneum*. Furthermore, a different research group reported that *S. officinarum* experienced two rounds of autopolyploidization and *S. spontaneum* experienced multiple polyploidization events independently after the two species separated from each other based on the distribution of shared TEs at the TOR and LFY haplotypes derived from *S. officinarum* and *S. spontaneum* genomes in the sugarcane hybrid R570 (Vilela et al., 2017). The authors found that most TE insertions occurred after the estimated divergence of *S. officinarum* and *S. spontaneum* at 2.5 to 3.5 mys and some of these insertions were restricted to *S. officinarum* haplotypes (Vilela et al., 2017). In this study, the authors did not find evidence of allopolyploidy shared between *Saccharum* and *Miscanthus* based on *Ks* values and shared TE insertions.

If *Saccharum* lineage originated from an allopolyploid ancestor followed by *Saccharum*-specific autopolyploidy, the distribution of *Ks* values of *S. officinarum* and *S. spontaneum* gene pairs should form two peaks, the older peak representing the divergence between the two distinct sub-genomes of the allopolyploid ancestor and the younger peak representing the divergence between the genes derived from the two sub-genomes via autopolyploidization. In our study, we detected a single sharp *Ks* peak at 0.02, which represents the divergence of *S. officinarum* and *S spontaneum* at 1.5 mys. Our result does not support the hypothesis of allopolyploidy occurred in the ancestor of *Saccharum* and *Miscanthus* followed by *Saccharum*-specific autopolyploidy.

Transposable elements form a large fraction of plant genomes. Although transposable element activity is tightly controlled in plant genomes by silencing or eliminating the TE copies, retrotransposition of TEs can be induced by stress (Wessler, 1996; Ito et al., 2016), tissue culture (Hirochika et al., 1996; Rhee et al., 2010), or events such as hybridization and polyploidy (Kashkush et al., 2002; Vicient and Casacuberta, 2017). Transposable element activation following polyploidy has been reported in numerous studies. Periodic bursts of centromeric LTR retrotransposon activity occurred after allopolyploidy through repeated formation of recombinants in maize genome (Sharma et al., 2008). Similarly, specific LTR retrotransposon families showed proliferation following autopolyploidy in the Buckler Mustard species complex (Bardil et al., 2015) and allopolyploidy in several other plant systems (Parisod et al., 2010; Senerchia et al., 2014). With the passage of time, TE insertions degenerate due to mutations, nested insertions, and deletions, making it difficult to identify shared insertions in diverged genomes. The half-life of LTR retrotransposons is shorter in smaller genomes such as *Arabidopsis* and rice and longer in large genomes such as wheat (Wicker and Keller, 2007). The half-life of LTR retrotransposons in rice, one of the smallest cereal genomes, was estimated at 4–6 my (Ma and Bennetzen, 2004; Zhang and Gao, 2017), which is longer than the estimated time of allopolyploidy in sugarcane at 3.8–4.6 mys (Kim et al., 2014). Surprisingly, most full-length LTR retrotransposon copies have inserted recently in both *S. officinarum* and *S. spontaneum*, long after the time of allopolyploidy (3.8 mys) proposed by Kim et al. (2014). In fact, of the 38 full-length retrotransposon elements identified in *S. officinarum* and 37 elements in *S. spontaneum*, none of them were older than 2.6 my and most had inserted in *S. officinarum* and *S. spontaneum* within the recent 1.6 and 0.9 my, respectively. Although a few full-length LTR retrotransposon insertions were shared by homologous chromosomes within *S. officinarum* and within *S. spontaneum*, no full-length elements were shared between *S. officinarum* and *S. spontaneum*. If retrotransposition was activated following allopolyploidy, a large number of young TEs should be identified in both *S. officinarum* and *S. spontaneum* genomes. A dearth of TE insertions shared by *S. officinarum* and *S. spontaneum* supports the latter hypothesis that two or more autopolyploidization events occurred independently in *S. officinarum* and *S. spontaneum* after their divergence. Contrary to earlier expectations, however, it is possible that retrotransposition was not activated following allopolyploidy in *Saccharum* or that the LTR insertions were purged from *Saccharum* genome rather quickly.

Although no shared full-length LTR retrotransposons were identified in *S. officinarum* and *S. spontaneum*, several remnants of shared TEs were identified based on unique TE junctions in *S. officinarum* and *S. spontaneum*. In general, the estimated number of nucleotide substitutions per site (*K*) between *S. officinarum* and *S. spontaneum* were much higher than those between homologous regions within *S. officinarum* and within *S. spontaneum*. Divergence time estimated using an intergenic region harboring a TE-junction shared by 3 So and 1 Ss BACs revealed that the *S. spontaneum* intergenic region was distant to those from the 3 homologous regions in *S. officinarum*. In addition, the same pattern of divergence was observed using the divergence (*Ks*) of a syntenic gene shared by all four BACs. Our result supports the latter hypothesis that *S. officinarum* experienced independent autopolyploidization events following its divergence from *S. spontaneum* (Vilela et al., 2017). However, we cannot exclude the possibility that high recombination and gene conversion may have homogenized the regions we examined from *S. officinarum* and *S. spontaneum*. Therefore, close examination of shared TEs at several other locations is warranted.

In summary, *S. officinarum* and *S. spontaneum* share a high degree of collinearity in genic regions. We did not find evidence of an early allopolyploidy in *Miscanthus*–*Saccharum* ancestor as proposed by Kim et al. (2014). The presence of many young LTR TEs, the absence of TEs closer to the proposed time of allopolyploidy, and high similarity of intergenic regions and a syntenic gene in at least 3 So BACs relative to the Ss BAC lend strong support to the hypothesis that *S. officinarum* and *S. spontaneum* experienced at least two rounds of independent polyploidizations in each lineage after their divergence from each other roughly 2 mys. The *S. officinarum* and *S. spontaneum* BAC libraries are a valuable resource for genomic studies of *Saccharum* and provide the foundation for identification of *S. spontaneum* and *S. officinarum* fractions in modern sugarcane genome. These BAC libraries can also be used for identification and characterization of targeted gene families, and for comparative and evolutionary genomics studies in sugarcane.

## Data Availability Statements

The assembled BACs have been deposited in GenBank under the accession numbers MH182499-MH182581 and KU685404-KU685417.

## Author Contributions

QY and RM designed the experiments, coordinated, and organized the all research activities. AS, JS, QL, RS, NR, KW, and JZ conducted the experiment and data analysis. AS and QY wrote the manuscript. All the authors read and approved the final manuscript.

## Conflict of Interest Statement

The authors declare that the research was conducted in the absence of any commercial or financial relationships that could be construed as a potential conflict of interest.

## Footnotes

1 http://www.lucigen.com

2 http://www.premierbiosoft.com/primerdesign/

3 http://www.hrt.msu.edu/uploads/535/78637/CRL_Scripts1.0.tar.gz

4 http://www.hrt.msu.edu/uploads/535/78637/alluniRefprexp070416.gz

5 ftp://ftpmips.helmholtz-muenchen.de/plants/REdat/

6 http://www.girinst.org/
