# Supplementary material for: Comparative Analysis of Homologous Sequences of Saccharum officinarum and Saccharum spontaneum Reveals Independent Polyploidization Events
Source: Front Plant Sci. 2018 Sep 25;9:1414. doi: 10.3389/fpls.2018.01414 (PMC6167920; doi:10.3389/fpls.2018.01414)
Supplement: Supplementary file 5 [file Image_1.PDF]

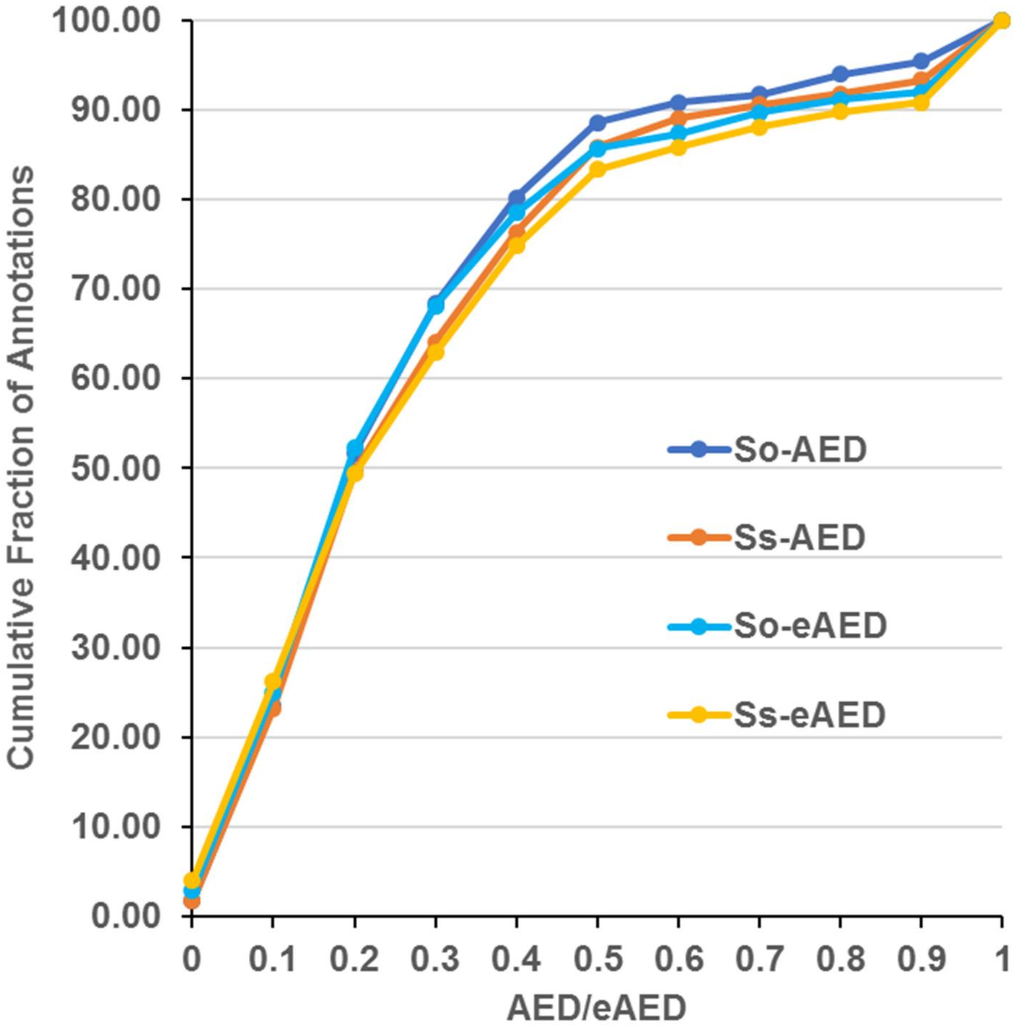

Supplementary Figure S1. Quality of gene model annotation in So and Ss BACs. The cumulative fraction of annotations (Y axis) are plotted against the AED scores (X asis). Approximately 86% of gene models in Ss BACs and 89% of gene models from So BACs had AED ≤ 0.5.
