# Supplementary material for: Comparative Analysis of Homologous Sequences of Saccharum officinarum and Saccharum spontaneum Reveals Independent Polyploidization Events
Source: Front Plant Sci. 2018 Sep 25;9:1414. doi: 10.3389/fpls.2018.01414 (PMC6167920; doi:10.3389/fpls.2018.01414)
Supplement: Supplementary file 6 [file Image_2.PDF]

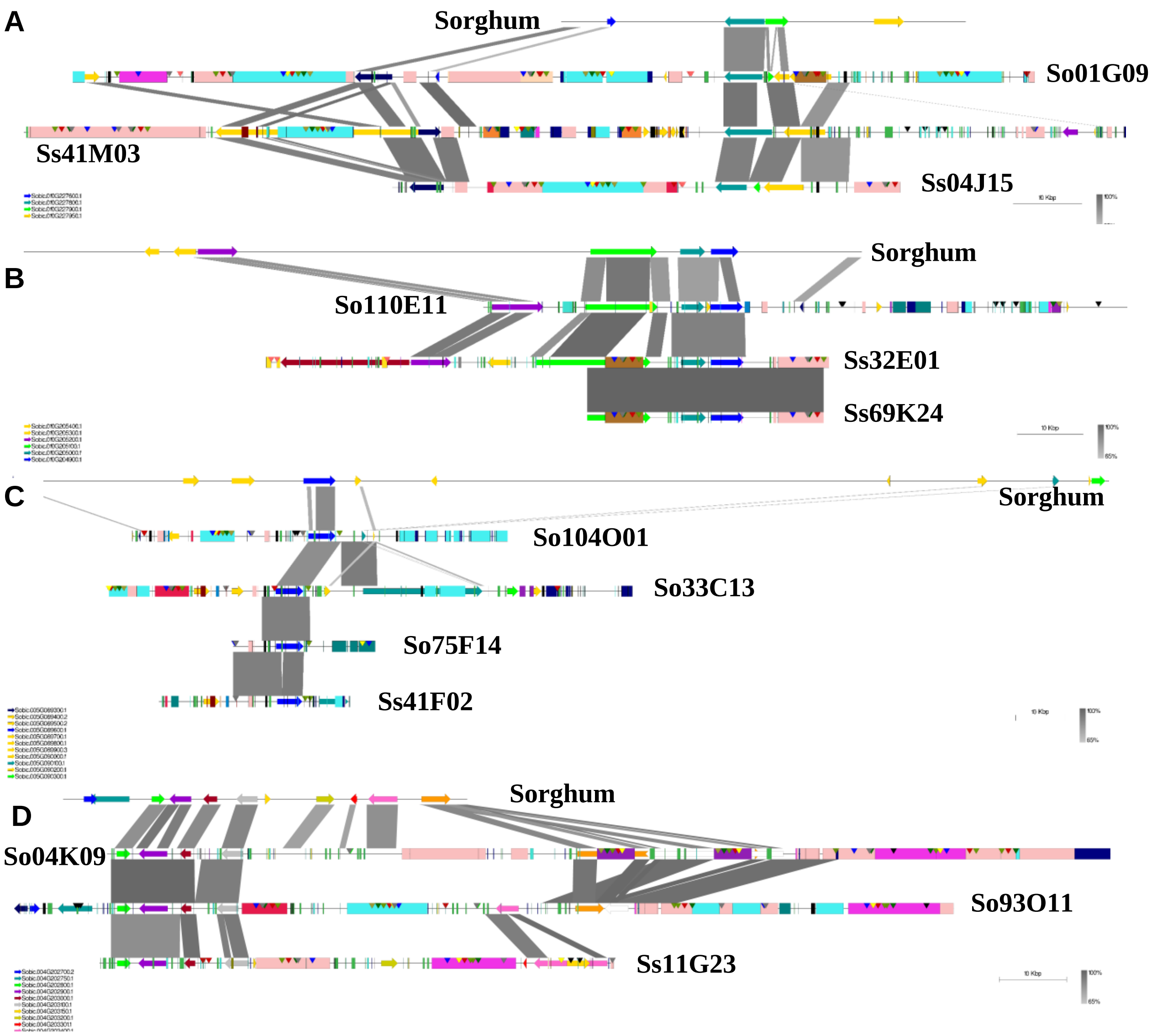

Supplementary Figure S2. Alignment of syntenic regions between *Saccharum* and sorghum and between *S. officinarum* and *S. spontaneum*. (A) So01G09/Ss41M03/Ss04J15; (B) So110E11/Ss32E01/Ss69K24; (C) So104O01/So33C13/So75F14/Ss41F02; (D) So04K09/So93O11/Ss11G23. The color-coded arrows represent genes, rectangles represent repeats, and conserved domains in transposable elements are represented by pointers. The blast similarity between annotated genic regions is shown by connectors in gray color gradient. A high degree of co-linearity is shared between *Saccharum* and sorghum and between *S. officinarum* and *S. spontaneum*. The large TEs are shared by homologous regions within the same species but not by the ones from different *Saccharum* species.
